# COGNAC: a web server for searching and annotating hydrogen-bonded base interactions in RNA three-dimensional structures

**DOI:** 10.1093/nar/gku438

**Published:** 2014-05-15

**Authors:** Mohd Firdaus-Raih, Hazrina Yusof Hamdani, Nurul Nadzirin, Effirul Ikhwan Ramlan, Peter Willett, Peter J. Artymiuk

**Affiliations:** 1 School of Biosciences and Biotechnology, Faculty of Science and Technology, Universiti Kebangsaan Malaysia, 43600 UKM Bangi, Malaysia; 2 Institute of Systems Biology, Universiti Kebangsaan Malaysia, 43600 UKM Bangi, Malaysia; 3 Department of Artificial Intelligence, Faculty of Computer Science and Information Technology, University of Malaya, 50603 Kuala Lumpur, Malaysia; 4 Information School, University of Sheffield, Western Bank, Sheffield S10 2TN, UK; 5 Department of Molecular Biology and Biotechnology, Krebs Institute, University of Sheffield, Western Bank, Sheffield S10 2TN, UK

## Abstract

Hydrogen bonds are crucial factors that stabilize a complex ribonucleic acid (RNA) molecule's three-dimensional (3D) structure. Minute conformational changes can result in variations in the hydrogen bond interactions in a particular structure. Furthermore, networks of hydrogen bonds, especially those found in tight clusters, may be important elements in structure stabilization or function and can therefore be regarded as potential tertiary motifs. In this paper, we describe a graph theoretical algorithm implemented as a web server that is able to search for unbroken networks of hydrogen-bonded base interactions and thus provide an accounting of such interactions in RNA 3D structures. This server, COGNAC (COnnection tables Graphs for Nucleic ACids), is also able to compare the hydrogen bond networks between two structures and from such annotations enable the mapping of atomic level differences that may have resulted from conformational changes due to mutations or binding events. The COGNAC server can be accessed at http://mfrlab.org/grafss/cognac.

## INTRODUCTION

**Figure 1.**
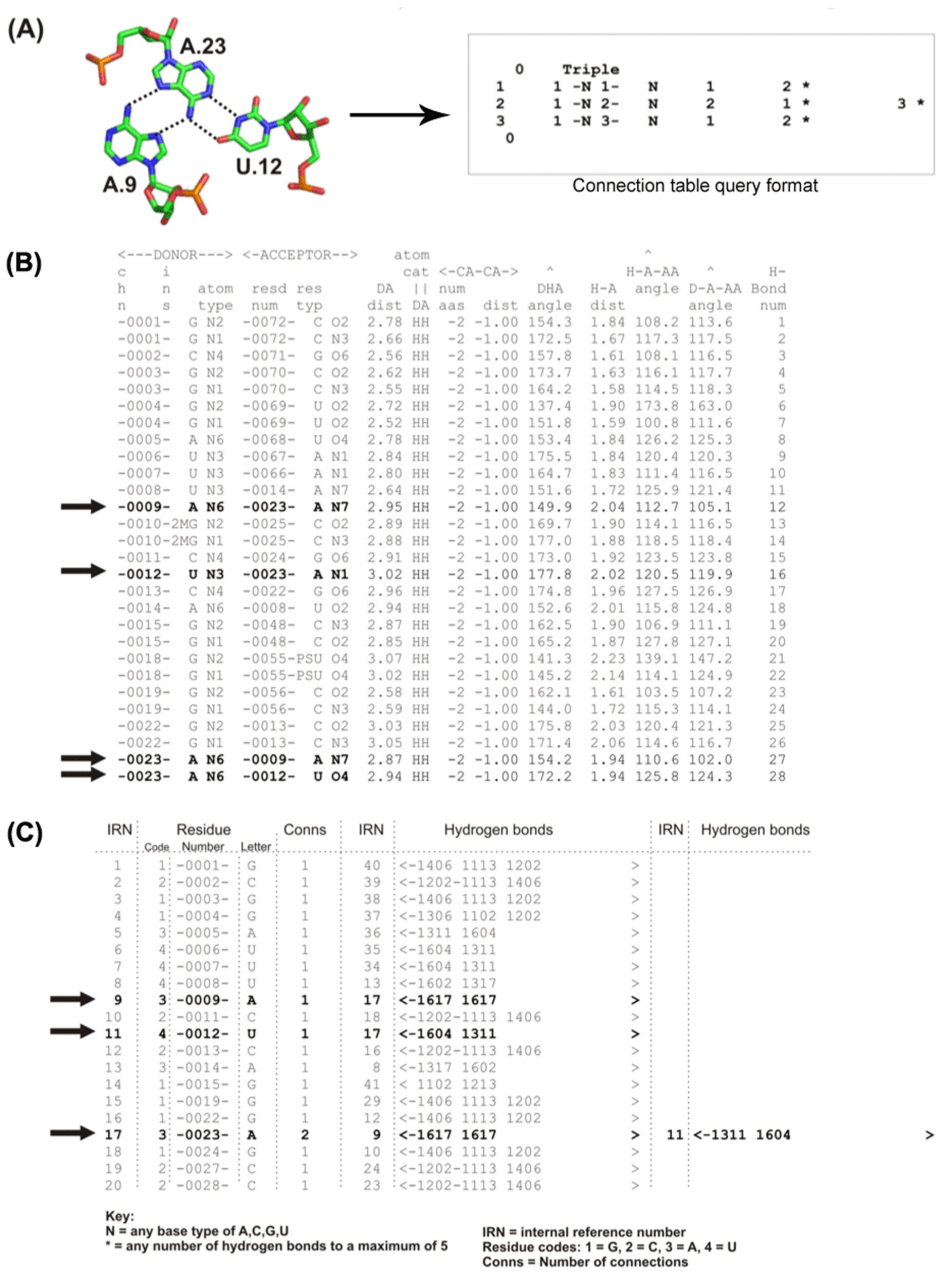
(**A**) Base triple interaction example A9.A23.U12 from the yeast tRNA^Phe^ structure (PDB ID: 6tna, left panel) and it's corresponding representation in a connection table (right panel). (**B**) Partial sample of HBPRED output file with the four hydrogen bonds for the triple in (A) highlighted in bold. (**C**) Partial sample of the connection table file after conversion from the format in (B) with the interactions for the AAU triple highlighted in bold. The highlighted data in (C) can be read as ‘base A9 (IRN = 9) is connected to one other base, A23 (IRN = 17), by two hydrogen bonds; base U12 (IRN = 11) is connected to one other base, A23, by two H-bonds; base A23 is connected to two other bases A9 and U12 by two hydrogen bonds each’. ‘−1604 1311’ can be read as ‘A23 N6 (1 = nitrogen) donor to U12 O4 (0 = oxygen) acceptor and U12 N3 donor to A23 N1 acceptor’.

The three-dimensional (3D) structures of complex ribonucleic acid (RNA) molecules are as crucial for their function as they are for proteins. The contribution by networks of hydrogen-bonded interactions towards structural stabilization have been well studied in RNA structures (1–5). Efforts have been made to annotate the 3D structures of RNA in order to analyze their functions. The majority of such structural annotations have focused on the alignment of RNA 3D structures to detect similarities in folding and sub-folding (6–8) and these approaches are not unlike those used in protein fold comparisons where an often used method involves superpositions of the atoms forming the structures’ backbones. Other programs such as NASSAM (9) and WebFR3D (10) analyze the spatial arrangements of the RNA bases in order to identify motifs. Lescoute and Westhof had previously explored the annotation of interaction networks for three-way junctions in folded RNAs (11). This annotation effort involved the mapping of base–base interaction networks using the nomenclature system previously proposed by Leontis and Westhof (12) that further complemented the earlier work by Gutell *et al.* (13). In the literature, investigations that specifically focused on analyzing the interactions of base triples (example in Figure 1A, left panel) had been carried out by Abu Almakarem *et al.* (14) using FR3D (15) and Firdaus-Raih *et al.* (16,17) using the computer program NASSAM (9). However, these previous studies have not specifically explored regions of the RNA 3D structures that involve tight clusters of interacting bases or the occurrences of unbroken networks of hydrogen-bond-mediated base interactions.

The hydrogen-bonded base interaction networks within an RNA structure can be thought of as two-dimensional (2D) networks. Previously, Gan *et al.* (18) described the use of planar graphs to represent RNA structural topology. In a similar way, the hydrogen-bonded interactions between bases can also be represented via the use of tree graphs. In such a tree representation, hydrogen bonds can be represented by the graph's edges and these bonds can be either single, bifurcated or multiple interactions. Labels can be added to these graphs, where each node can represent one of the four bases or simply a ‘wild-card’ to represent any of the bases. The edges can also be similarly labeled in such a way that an edge can represent a specific hydrogen bond, a particular number of hydrogen bonds or any number of hydrogen bonds. The information within these labeled graphs can be contained within the tabular data structure of a connection table (Figure 1A, right panel). A graph theoretical algorithm can then be employed to study relationships between the connection tables (19).

As various structure-dependent functional RNA molecules are discovered and more RNA structures become available in the Protein Data Bank (PDB) (20), a computational tool that can search, annotate and provide comparisons between large numbers of structures is expected to be a useful utility for RNA biologists in general. In this article, we describe a server that is able to map hydrogen bonds between RNA bases and from this accounting of such interactions, allow for the annotation of RNA base interaction clusters that may potentially be structural motifs; in addition, it may also yield potential insights regarding the functional or structure stabilization roles for clusters of interacting bases.

## PROGRAMS AND METHODS

The COGNAC (COnnection tables Graphs for Nucleic ACids) server consists of three major components: the hydrogen bond data generating program, the graph theoretical COGNAC algorithm and the visual analysis component. The hydrogen bond generating program, HBPRED (Hydrogen Bonding Predictor) , calculates the formation of a hydrogen bond between donor and acceptor atoms using the parameters previously used in HBPLUS (21). The HBPRED outputs (Figure 1B) are then converted into a connection table format (Figure 1C) that is searchable by the main COGNAC program. HBPRED generates the hydrogen bonding data for user provided structures as well as for structures in the internal database that were sourced from the PDB. An example of a base triple is provided in Figure 1A where a base triple with four hydrogen bonds is part of a transfer RNA structure (PDB ID: 6tna). The HBPRED program generates a list of all possible hydrogen bond interactions in the structure (Figure 1B) which is then converted into a connection table (Figure 1D). A query in connection table format for the triple (Figure 1B) that is processed through COGNAC is then able to identify the subgraph of the query (Figure 1A, right panel) in the larger connection table for the whole structure (Figure 1C).

**Figure 2.**
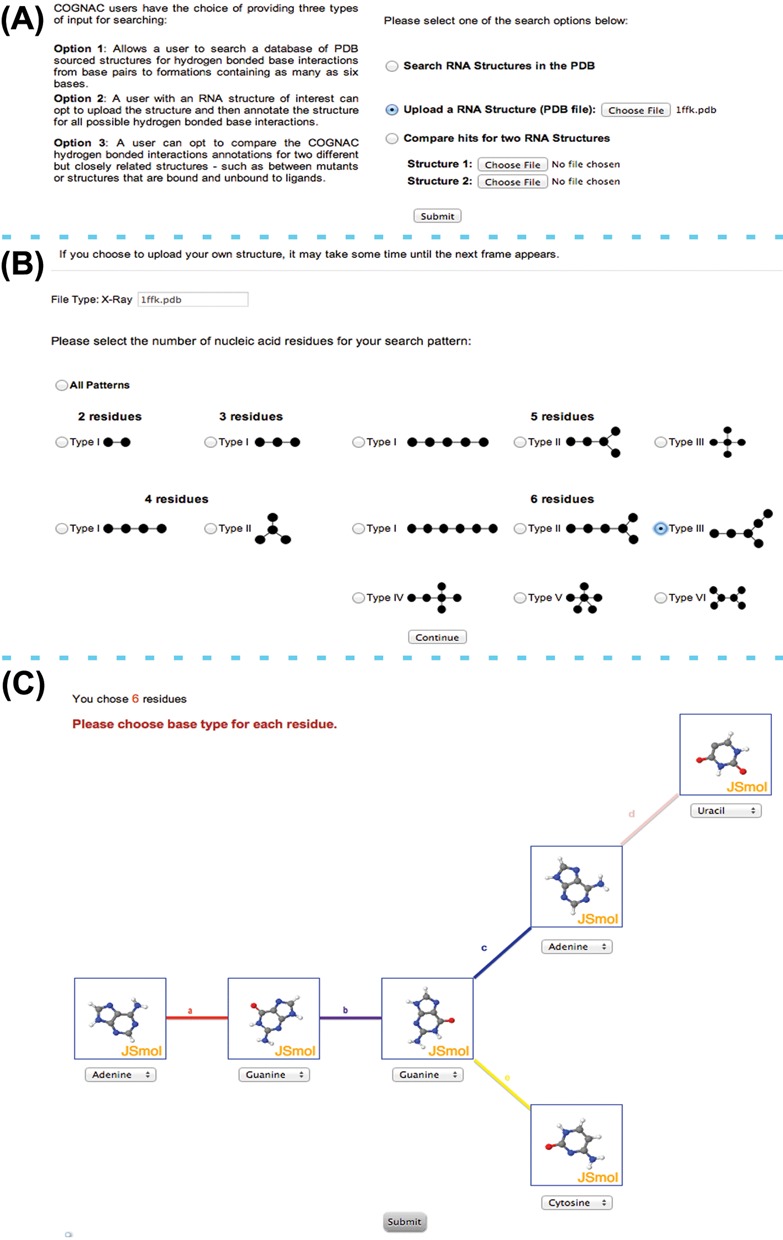
Input interface for COGNAC where: (**A**) users can initially select between three types of search options; the example presented shows the option to upload a user provider query being selected. (**B**) A follow-up interface proceeding the initial options prompts users to select the type of interaction to search for based on the representative tree graphs for the interaction of interest; the example presented shows the selection of a sextuple arrangement. (**C**) An additional search specification option where users can define the bases for the query involved in the sextuple arrangement that has been selected in (B); users have the option of having the query design facilitated by a mini JSmol window depicting the molecular structure for each base.

The COGNAC program is a graph theoretical implementation of the Ullmann subgraph isomorphism (22) that solves the problem of matching a query connection table graph representation to its matching subgraph. The query submission interface allows for the searching and annotation of structures within an internal database or for user provided RNA structures (Figure 2A). The base interaction possibilities for COGNAC search patterns were enumerated and are represented as tree graphs that range from single possibilities for pairs and triples to six possible unique interaction arrangements for sextuples (Figure 2B). The query for a COGNAC search consists of the bases represented as the graph nodes and the edges representing the number of connections (Figure 1B), which in this case refers to the number of hydrogen bonding interactions to each base. The number of hydrogen bonds represented by the edges can be left unspecified with a minimum of one bond. The nodes representing the bases can be pre-determined as a specific base or left as a wildcard base representing any of the base options specified in the base library, which includes several modified bases such as pseudouracil.

## INPUT AND OUTPUT

### Input description

The COGNAC query interface allows a user to submit three types of search options (Figure 2A). The first option enables a user to search a pre-set database of RNA chain containing structures that were sourced from the PDB. The database is updated on a monthly basis with the current version reported in this paper compiled on 12 March 2014 and contains 1778 files with structure resolutions >3.5 Å. The second option allows a user to upload a PDB formatted structure of interest while the third search option provides the user with the capability to search two PDB formatted structures simultaneously, thus allowing for the differences between the hydrogen bonds formed within the two structures to be investigated. For structures that were generated using nuclear magnetic resonance spectroscopy (NMR) data, the user will be prompted to select only one model for analysis at a time. The program will also automatically remove all hydrogen atoms in NMR structures and thus these atoms will no longer be visible when the hits are visualized using either the Jmol or JSmol viewers.

Once the initial type of search has been selected, a new interface appears which provides the user with the option of either (i) searching all patterns of base pairs to base sextuples that have been enumerated or (ii) searching for a specific base-to-base connection that ranges from a pair to a sextuple (Figure 2B). Upon selection of the type of representative graph(s) for the connection table, another interface appears prompting the user to provide a specific base as the graph's node or to leave the base as a wildcard option (Figure 2C). This interface uses JSmol (http://wiki.jmol.org) windows to enable users to orientate the bases in order to facilitate a visual aided approach toward envisioning possible hydrogen bonding interactions that may occur. However, the orientations of the bases in the JSmol windows are not used as a parameter for the search because only information on the base type is required for the searches.

### Output description

**Figure 3.**
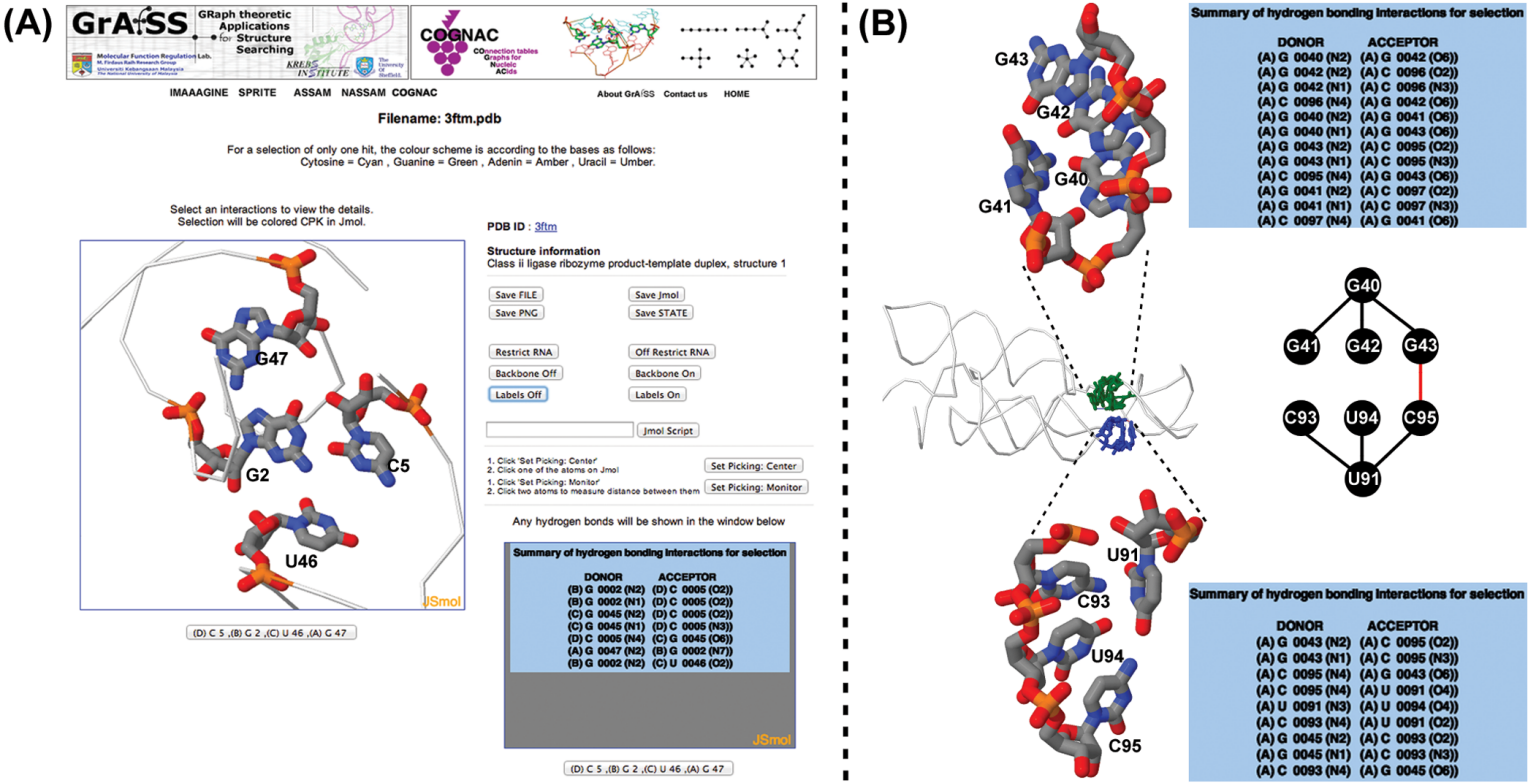
Examples of COGNAC output: (**A**) the molecular visualization interface using JSmol (or Jmol) with basic information on the structure, external links to the PDB entry and a listing of the hydrogen bonds involved in the interaction network which in this case is a base quadruple; (**B**) two matches for a type II quadruple (refer to Figure 2B) search tree query in a lysine riboswitch structure—a GGGG quadruple (center panel, in green) and a CUCU quadruple (center panel, in blue) that upon further scrutiny is interconnected (denoted by red line) to form an octuple base interaction (center panel).

COGNAC searches for all three input options will first output a listing of the number of matches to a particular type of interaction (tree) queried. The raw output is filtered for redundant hits that arise due to the searches being independent of sequence order thus resulting in the same hits being retrieved for the same interaction for the different sequence order possibilities. The filtered output is presented to the user as a summary of the number of hits for a particular tree query or queries. Selecting the output for a specific tree graph then results in a list that provides information regarding the structure in which a match is found and a listing of the hydrogen bonds involved in a particular match. A user can then select a particular hit for further inspection using the built-in molecular visualization interfaces (Figure 3). Molecular visualization can be carried out using either the Jmol or JSmol (http://wiki.jmol.org) options. Jmol, a Java plugin, is noticeably more responsive at handling large structures such as ribosomal RNA when compared to JSmol. However, some operating systems have been known to block the use of Java due to security concerns and Jmol use may also be inconvenient to some users due to the requirement of needing Java installed for the browser. The JSmol viewers on the other hand will work directly inside the browsers without any additional installation requirements. Both the Jmol and JSmol windows allow for in browser analysis of the interactions and a further interaction by interaction inspection of the hits. Each molecular visualization window is accompanied by a detailed listing of the hydrogen bond interactions computed by HBPRED (Figure 3). Additionally, the hydrogen bonds are by default drawn into the visual display for each hit with the respective bond lengths labeled. The server also generates the 2D structure representations from the HBPRED data using the RNAfold program from the ViennaRNA Package (23). For the comparison of similar structures, a listing that highlights the differences between two structures is provided with a further visualization option of comparing the structures in adjacent synchronized Jmol or JSmol windows.

## DISCUSSION AND CASE STUDIES

Currently, several web servers such as WebFR3D (10), NASSAM (9), FASTR3D (24), FRASS (25), SARA (26), ARTS (7) and RNA FRABASE (27) are available for RNA structural analysis. These servers employ different approaches, require different input types and produce different types of output. To our knowledge, the most similarly intended programs to COGNAC are the NASSAM and WebFR3D servers. The program WebFR3D (10) allows two modes of searching a database: a geometric search that finds all motifs similar to a user-specified query structure; and a symbolic search that finds all sets of nucleotides making user-specified interactions. The searchable structures for WebFR3D are currently limited to those already in the database because the program does not appear to allow for user-supplied structures to be analyzed.

The NASSAM server (9) takes a PDB formatted structure and searches it for a specific 3D arrangement of the RNA bases against its pattern database. Such an approach requires prior knowledge of the 3D arrangement for an interaction or motif and is therefore restricted when posed with generic queries that require, for example, all triples or quintuples in a structure to be listed. COGNAC, although able to search for triples that are defined by a 3D arrangement, does not find arrangements that are outside of the hydrogen-bonding parameters that have been specified. Although NASSAM has demonstrated a high degree of flexibility in extrapolating search patterns towards searching for novel orientations, it is still not able to accept a more general and less restrictive wildcard type query where, for example, the type and orientation of one or all the bases are not specifically defined. Searches that incorporate such wildcard options are advantageous for both the searching of novel base orientations and the searching of large hydrogen bonded base-base interaction clusters. Due to the obvious difficulties involved in theoretically predicting base interaction orientations for clusters in excess of three bases, the COGNAC querying capability presents an ideal solution to resolve this issue.

An accounting of the current database used revealed that sextuple interactions represented by the tree patterns sextuple types IV and V in Figure 2B have yet to be found in any existing reasonably high resolution X-ray crystallographic RNA structures in the PDB. This is not unsurprising for the type V sextuple pattern due to the number of interactions the central node is required to have and thus may probably not be sterically or chemically possible. Searching with COGNAC was able to retrieve the expected extended planar interactions consisting of mainly triples and quadruples (Figure 3A). Interactions that included bases orientated off plane to each other (Figure 3B) that were still able to form hydrogen bonds had been observed for the networks involving more than three bases. In the structure of a lysine riboswitch (PDB ID: 4erl, 4erj) (28), two adjacent quadruples were detected by COGNAC (Figure 3B, center panel). These adjacent quadruples were composed of different bases with one being an all guanine quadruple (Figure 3B, top panel) and the other comprising of two cytosines and two uracils (Figure 3B, bottom panel). Despite the base composition difference, both quadruples consisted of a similar arrangement where one base is connected to three other bases. This observation is also interesting due to the fact that the two separate quadruples are interconnected and therefore are components of a larger octuple interaction (Figure 3B, center panel).

## SUMMARY

The COGNAC web server, when deployed to annotate existing RNA structures, is expected to further complement existing resources by bridging the gap between the complexity of RNA tertiary interactions to highly organized annotation schemes and 2D representations. From the testing and comparison of the currently available services, it is clear that the information and types of outputs provided by the different web servers do not overlap. To our knowledge, there are also no available servers that enable a user to compare the differences in the hydrogen bond interactions present between two very similar structures. This is an especially useful feature when attempting to detect the minute differences that can occur between RNA structures in different states such as between mutants and wild-types or between ligand bound and unbound states. In fact, the WebFR3D, NASSAM and COGNAC services can be seen as complementary to each other and can provide an array of tools to address different questions that can be posed for an RNA structure.

## ACCESSION NUMBERS

PDB IDs: 6tna, 4erl and 4erj.
